# Age-Dependent, Subunit Specific Action of Hydrogen Sulfide on GluN1/2A and GluN1/2B NMDA Receptors

**DOI:** 10.3389/fncel.2017.00375

**Published:** 2017-11-24

**Authors:** Aleksey V. Yakovlev, Evgeniya D. Kurmasheva, Yevheniia Ishchenko, Rashid Giniatullin, Guzel F. Sitdikova

**Affiliations:** ^1^Department of Human and Animal Physiology, Institute of Fundamental Medicine and Biology, Kazan Federal University, Kazan, Russia; ^2^Laboratory of Molecular Pain Research, A. I. Virtanen Institute for Molecular Sciences, University of Eastern Finland, Kuopio, Finland; ^3^Laboratory of Neurobiology, Institute of Fundamental Medicine and Biology, Kazan Federal University, Kazan, Russia

**Keywords:** hydrogen sulfide, hippocampal slices, NMDA receptors, GluN1/2A, GluN1/2B, redox modulation, adenylate cyclase

## Abstract

Hydrogen sulfide (H_2_S) is an endogenously produced neuroactive gas implicated in many key processes in the peripheral and central nervous system. Whereas the neuroprotective role of H_2_S has been shown in adult brain, the action of this messenger in newborns remains unclear. One of the known targets of H_2_S in the nervous system is the N-methyl-D-aspartate (NMDA) glutamate receptor which can be composed of different subunits with distinct functional properties. In the present study, using patch clamp technique, we compared the effects of the H_2_S donor sodium hydrosulfide (NaHS, 100 μM) on hippocampal NMDA receptor mediated currents in rats of the first and third postnatal weeks. This was supplemented by testing effects of NaHS on recombinant GluN1/2A and GluN1/2B NMDA receptors expressed in HEK293T cells. The main finding is that NaHS action on NMDA currents is age-dependent. Currents were reduced in newborns but increased in older juvenile rats. Consistent with an age-dependent switch in NMDA receptor composition, in HEK239T cells expressing GluN1/2A receptors, NaHS increased NMDA activated currents associated with acceleration of desensitization and decrease of the deactivation rate. In contrast, in GluN1/2B NMDA receptors, which are prevalent in newborns, NaHS decreased currents and reduced receptor deactivation without effect on the desensitization rate. Adenylate cyclase inhibitor MDL-12330A (10 μM) did not prevent the age-dependent effects of NaHS on NMDA evoked currents in pyramidal neurons of hippocampus. The reducing agent dithiothreitol (DTT, 2 mM) applied on HEK293T cells prevented facilitation induced by NaHS on GluN1/2A NMDA receptors, however in GluN1/2B NMDA receptors the inhibitory effect of NaHS was still observed. Our data indicate age-dependent effect of H_2_S on NMDA receptor mediated currents determined by glutamate receptor subunit composition. While the inhibitory action of H_2_ on GluN1/2B receptors could limit the excessive activation in early age, the enhanced functionality of GluN1/2A receptor in the presence of this gasotransmitter can enlarge synaptic efficacy and promote synaptic plasticity in adults.

## Introduction

Hydrogen sulfide (H_2_S) is a member of gasotransmitters family involved in the regulation of neuronal plasticity, excitability and neurotransmitter release in the peripheral and central nervous system (Abe and Kimura, 1996; Wang, 2012; Gerasimova et al., 2015; Yakovlev et al., 2017). H_2_S has been demonstrated to induce long-term potentiation (LTP) in the hippocampus (Abe and Kimura, 1996), modulate neuronal excitability of the subfornical organ and the nucleus of the solitary tract (Kuksis et al., 2014; Kuksis and Ferguson, 2015; Malik and Ferguson, 2016) and mediate central inhibition of the respiratory system (Chen et al., 2013). Endogenous H_2_S in the brain is produced by cystathionine β-synthase (CBS) from cysteine or L-homocysteine. In addition, H_2_S can also be produced from cysteine by D-amino acid oxidase (DAO) or 3-mercaptopyruvate sulfurtransferase (3-MST) in combination with cysteine aminotransferase (CAT; Shibuya et al., 2009, 2013; Kimura, 2014). It has been shown, that H_2_S produces anti-inflammatory, antioxidant and antiapoptotic effects in glial and neuronal cells (Lee et al., 2010; Kamat et al., 2015). H_2_S rescues behavioral and memory deficits in neurodegenerative diseases such as Alzheimer’s disease (AD), Parkinson’s disease (PD), and vascular dementia (Eto et al., 2002; Hu et al., 2010). Furthermore, H_2_S showed a protective effect in the impairment of the spatial memory caused by acute stress (He et al., 2017). In neonatal brain H_2_S abolished interictal-like events induced by bicuculline preventing enhanced neuronal excitability typical to early hippocampal networks (Yakovlev et al., 2017).

N-methyl-D-aspartate (NMDA) receptors are one of the targets of H_2_S in the brain. It has been reported that H_2_S specifically potentiates the activity of NMDA receptors and facilitates the induction of hippocampal LTP (Abe and Kimura, 1996). However, we recently demonstrated that in neonatal hippocampal slices H_2_S is able to decrease NMDA receptor mediated currents in pyramidal neurons of the CA3 region (Yakovlev et al., 2017). This variability could reflect the differences in subunit composition of NMDA receptors of neonatal and juvenile/adult animals. Thus, in hippocampus, during the first postnatal week NMDA receptors are mainly composed by GluN1/2B subunits whereas in the adult brain there is a contribution of GluN1, GluN2A and GluN2B subunits (Liu et al., 2004; Chang et al., 2009).

The aim of the present study was to compare the effects of H_2_S donor-sodium hydrosulfide (NaHS) on the NMDA activated currents in pyramidal neurons of rat hippocampus during first and third postnatal weeks. We further employed to clarify subunit specific effects of NaHS on NMDA receptors using recombinant GluN1/2A and GluN1/2B receptors expressed in HEK293T cells. The role of adenylate cyclase activity and disulfide bonds in the effects of H_2_S was also studied.

## Materials and Methods

### Slice Preparation

Hippocampal slice preparation was performed using *Wistar* rats of two age groups—postnatal days P 3–7 and P 18–26 (P0-date of birth). The work has been carried out in accordance with EU Directive 2010/63/EU for animal experiments and all animal-use protocols were approved by the Local Ethical Committee of Kazan Federal University. Animals were anesthetized with isoflurane or were subjected to cryoanesthesia before decapitation. After isolation, the rat brains were placed into a cooled oxygenated artificial cerebrospinal fluid (ACSF) of the following composition (in mM): NaCl 126; KCl 3.5; CaCl_2_ 2.0; MgCl_2_ 1.3; NaHCO_3_ 25; NaH_2_PO_4_ 1.25 and glucose 10 (pH 7.4). Horizontal slices of brain (400 μm thick) were cut using a HM 650 V vibratome (Microm International, Germany) and kept 1 h before use in oxygenated ACSF at room temperature. Individual slices were then transferred to the recording chamber where they were fully submerged and superfused with oxygenated ACSF (33–32°C, 3–4 ml/min).

### HEK293T Cell Line Maintaining and Transfection

HEK293T cell culture line was routinely grown as previously described (Fabbretti et al., 2004). Before transfection cells were placed for 4–12 h in 35 mm dishes (10 × 10^4^ cells per dish) on 7 mm poly-L-lysine (0.2 mg/ml) coated glass cover-slips in Dulbecco’s modified medium (DMEM, Sigma-Aldrich, St. Louis, MO, USA) supplemented with 10% fetal bovine serum (Gibco Invitrogen,Waltham, MA, USA) and kept in an incubator at 37°C in 5% CO_2_. For transfection we used 2 μg total DNA and 4 μl FuGene per well (Promega, Madison, WI, USA). Mammalian expression vectors were obtained from Dr. J. W. Johnson (University of Pittsburgh, Pittsburgh, PA, USA). The ratio of cDNA used was 10 EGFP: 30 GluN1: 60 GluN2 (A or B). After cells were incubated for 6–8 h, DMEM with transfection mix was replaced with fresh DMEM containing 200 μM DL-2-amino-5-amino-5-phosphono-valeric acid to prevent NMDA receptor mediated excitotoxicity and placed into the incubator (at 37°C in 5% CO_2_). Cells were used for electrophysiological recording 24–48 h after transfection.

### Electrophysiological Recordings

Patch-clamp recordings from the CA3 pyramidal neurons of hippocampus were performed in whole-cell configuration. Currents were amplified using an Axopatch 200B amplifier (Axon Instruments, Whipple Rd Union City, CA, USA) with a gain of 5 mV/pA and a band pass of 0–2 kHz. The pipette solutions contained (in mM): Cs methanesulfonate 135; CaCl_2_ 0.1; EGTA 1; HEPES 10; NaATP 2 and NaGTP 0.4 (pH 7.25), osmolarity 290 mOsm; pH adjusted to 7.3 with CsOH. Patch pipettes had a resistance of 4–8 MΩ. NMDA currents were elicited by the local application of 100 μM NMDA + 30 μM glycine in the absence of Mg^2+^ ions. In all experiments, we monitored a rundown of NMDA evoked currents and administered the drugs only after the NMDA currents were fully stabilized. Neurons with a stable access resistance (R_a_ ≤ 15 MΩ; <±20% change during the recording period) were used for subsequent analysis.

HEK293T cells on coverslips were perfused with bath solution contained (in mM): NaCl 140; KCl 5; CaCl_2_ 1; HEPES 10 at pH 7.4. Patch-pipette solution had the following composition (in mM): CsCl 140; 10 EGTA 10; HEPES 10; NaATP 4. The pH was adjusted to 7.4 with CsOH. Experiments were performed at room temperature (23–25°C). Whole-cell patch clamp recordings of membrane currents of HEK293T cells expressing recombinant GluN1/2A or GluN1/2B receptors were performed using an EPC-9 amplifier operated by HEKA PatchMaster software (HEKA Electronic, Germany). The liquid junction potentials for Cs methanesulfonate based solution was 9.9 mV and for CsCl based pipette solution −11.9 mV, that were corrected offline for all data analysis.

We measured the peak amplitude of NMDA currents and the area under each trace by integration after zeroing the baseline. Total charge transfer during agonist application was calculated as the integrated current per time in micro or nano-Coulombs (μC or nC; Bolton et al., 2013). NMDA receptor desensitization was measured by fitting a single exponential function to current decays (Sibarov et al., 2016) using OriginPro 9.1 (Microcal, Northampton, MA, USA). We also measured the peak current (I_pk_) and steady state current (I_ss_) amplitudes which provided NMDA current desensitization as a ratio I_ss_/I_pk_ (Borschel et al., 2012). The current traces during deactivation were fitted with a single exponential function (Talukder et al., 2011). Experiments on GluN2A or GluN2B type NMDA receptors were always carried out on the same day with the same set of solutions. Recordings on cells expressing different subtypes were interleaved, hence duration after transfection and time-dependent effects on drug potency were ruled out.

### Chemicals

Chemicals used were: NMDA (50–100 μM), glycine (Gly, 30 μM), bicuculline (10 μM), 6-cyano-7-nitroquinoxaline-2, 3dione (CNQX, 10–40 μM), d-2-Amino-5-phosphopentanoate (d-APV, 40 μM), CGP55845 (CGP, 2 μM), MDL-12330A (10 μM), dithiothreitol (DTT, 2 mM; Sigma-Aldrich, St. Louis, MO, USA). TTX (1 μM) was purchased from Alomone labs (Jerusalem, Israel). NaHS (Sigma-Aldrich, St. Louis, MO, USA) was used as a source of H_2_S. In solution this compound dissociates to give HS^−^ which associates with H^+^ to produce H_2_S. At 37°C 14% of total sulfide is present as H_2_S calculated from the Henderson-Hasselbalch equation (Whitfield et al., 2008; Sitdikova et al., 2014). In our experiments NaHS was used in a concentration of 100 μM which yields about 14 μM H_2_S in the perfusion system which constantly flows to the recording chamber. Due to volatilization the H_2_S concentration further decreases by 50% of the initial level which amounts to approximately 7 μM H_2_S in the perfusate (Sitdikova et al., 2014). In addition, H_2_S is quickly bound to slice preparation tissue and undergoes oxidation, as it was shown in the plasma (Whitfield et al., 2008), which further decreases the effective H_2_S concentration. Stock solutions of NaHS were prepared immediately before each experiment and kept hermetically sealed in a dark place. In used concentration (100 μM) NaHS did not change a pH of extracellular solution.

### Local Drug Application

NMDA receptor mediated currents in pyramidal neurons of hippocampus were evoked by local application of 100 μM NMDA with 30 μM Gly by pressure (5–10 psi, duration 5 s) through a glass pipette with a pneumatic picopump (PC-820, WPI, Worcester, MA, USA). The pipette was located at a distance of about 50–250 μm from the neuronal soma. In HEK293T cells local application of solutions on GFP-positive cells was performed via a multi-barrel rapid perfusion system (Rapid Solution Changer RSC-200; BioLogic Science Instruments, Grenoble, France). In the experiments on hippocampal slice preparations GABA_A_ and GABA_B_ receptors were inhibited by bicuculline (10 μM) and CGP (2 μM), respectively whereas the α-amino-3-hydroxy-5-methyl-4-isoxazolepropionic acid (AMPA)/kainate receptors were inhibited by CNQX (15 μM).

### Data Analysis

Normality of sample data was evaluated with the Shapiro-Wilk test and for equal variances using F-test Origin Pro software (OriginLab Corp., Northampton, MA, USA). As agonist and agonist + drug treatments were always obtained from the same cell the paired Wilcoxon signed ranks test (*W*-test) was used to evaluate statistically significant differences between the parameters in control and treatment. Differences were considered as statistically significant at *p* ≤ 0.05; *n* indicates the number of neurons or cells, *N*-number of animals. Data are presented as mean ± SEM text.

## Results

### Age-Related Effects of NaHS on NMDA Evoked Currents in Rat Hippocampal Neurons

It is known that the subunit composition of NMDA receptors in rodent hippocampus is changing during postnatal development (Chang et al., 2009). GluN2B subunits have a maximal peak of expression during the first postnatal week (P 7–10). GluN2A subunit expression gradually increases after birth and reaches a maximum level after the second postnatal week (Chang et al., 2009; Paoletti, 2011). We therefore used two groups of animals divided by age: neonatal (P 3–7) and juvenile (P 18–26). Whole cell recordings from pyramidal neurons were performed at a holding potential of −60 mV and 100 μM NMDA + 30 μM glycine was locally applied from the puff pipettes. The membrane capacitance of pyramidal neurons in neonatal animals was 47 ± 3 pF (*n* = 15, *N* = 12) and of juvenile animals 98 ± 11 pF (*n* = 12, *N* = 8). The input resistance (R_in_) of pyramidal neurons from newborn animals was 1.3 ± 0.2 GOm and in juvenile animals −0.4 ± 0.1 GOm.

**Figure 1:**
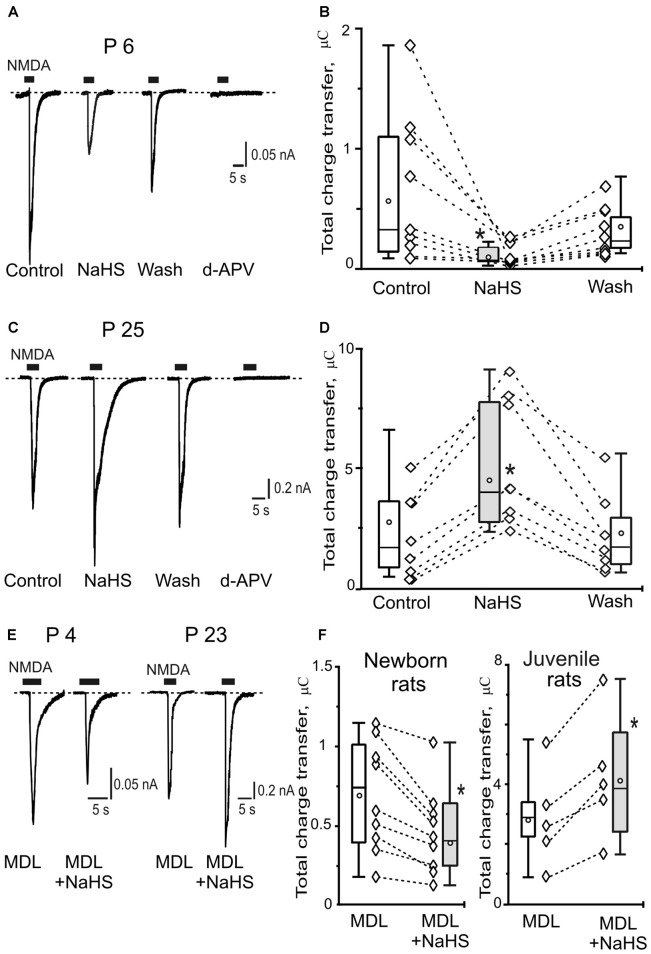
Age-dependent effects of sodium hydrosulfide (NaHS) on N-methyl-D-aspartate (NMDA) evoked currents in CA3 area of rat hippocampus. Representative current traces recorded from pyramidal neurons at a holding potential −60 mV activated by application of 100 μM NMDA + 30 μM glycine (5 s, black bars) in control, in the presence of 100 μM NaHS, after washout and after inhibition of NMDA receptors by d-2-Amino-5-phosphopentanoate (dAPV; 40 μM) at the age P6 **(A)** and at the age P25 **(C)**. Statistical plot of NMDA induced total charge transfer in control, in the presence of NaHS and after washout in pyramidal neurons of neonatal (**B**; *n* = 9, *N* = 6) and juvenile rats (**D**; *n* = 8, *N* = 6). Each pair of connected diamonds corresponds to an individual neuron. The mean values of boxplots are shown by white circles, whiskers—minimal and maximal values. **(E)** Representative current traces recorded from pyramidal neurons activated by application of 100 μM NMDA + 30 μM glycine (5 s, black bars) after incubation with the inhibitor of adenylate cyclase MDL-12330A (MDL; 10 μM) and in the presence of MDL+NaHS at the age P4 and P23. **(F)** Statistical plot of NMDA induced total charge transfer in the presence of MDL and MDL+NaHS in pyramidal neurons of neonatal (*n* = 9; *N* = 6) and juvenile (*n* = 5; *N* = 4) rats. Each pair of connected circles corresponds to individual neurons. Boxes indicate 25–75 percentiles in control (white) and in NaHS (gray), black line—median, the circle inside—mean value, whiskers—minimal and maximal values, *p < 0.05, paired *W*-test.

In hippocampal slices of newborn animals the local application of NMDA + Gly induced inward currents with an average amplitude of 0.4 ± 0.07 nA and a total charge transfer of 0.6 ± 0.2 μC (*n* = 9, *N* = 6). The inward currents were inhibited by the NMDA antagonist d-APV (Figure 1A). Preliminary application of NaHS for 5 min (100 μM) induced the robust decrease of NMDA receptor mediated currents (Figure 1A). The peak amplitude of NMDA induced currents decreased to 0.1 ± 0.04 nA (34 ± 18% of control; *p* = 0.007). The total charge transfer decreased to 0.1 ± 0.04 μC (34 ± 9% of control; *p* = 0.03; Figure 1B). The effects of NaHS on NMDA evoked currents were not fully recovered. After 15 min of washout with control solution the total charge transfer was 0.3 ± 0.08 μC (70 ± 11% of control; *p* = 0.04; Figures 1A,B).

In juvenile animals application of NMDA + Gly induced inward currents in hippocampus neurons with an average peak amplitude of 1.3 ± 0.5 nA (*n* = 8, *N* = 6) and a total charge transfer of 2.5 ± 0.7 μC (*n* = 8; Figures 1C,D). Preliminary bath application of NaHS (100 μM) increased the peak amplitude to 2.1 ± 0.6 nA (146 ± 18% of control; *p* = 0.02) and the total charge transfer of NMDA evoked currents was enhanced to 4.5 ± 1.3 μC (185 ± 22% of control; *p* = 0.01; Figure 1D). The NaHS effect was fully recovered after 15 min of washout with control solution and the total charge transfer returned to 2.1 ± 0.7 μC (104 ± 16% of control; *p* = 0.6; Figures 1C,D). The NMDA evoked currents were completely blocked by d-APV (40 μM; Figure 1C). Thus, NaHS induced opposite effects on NMDA activated currents in hippocampal pyramidal neurons in newborn and juvenile rats which can be explained by a developmental shift in NMDA receptor composition.

### Age-Dependent Effects of NaHS Were Not Prevented by the Inhibition of Adenylate Cyclase

It is known that H_2_S is able to increase cAMP production in primary neurons from cerebral cortex and cerebellum, as well as in glial cells (Kimura, 2000), suggesting the role of cAMP/protein kinase A (PKA) pathway in positive modulation of NMDA receptor functions by H_2_S. The inhibitor of adenylate cyclase MDL-12330A (10 μM) induced a reduction of NMDA evoked currents in the hippocampal neurons of immature animals (from 0.82 ± 0.07 to 0.55 ± 0.1 *n* = 3, *N* = 3; *p* = 0.03) and did not change NMDA-evoked currents in pyramidal neurons of juvenile animals (from 2.3 ± 1.0 to 2.7 ± 1.2; *n* = 3, *N* = 3; *p* = 0.06; data not shown). These results agreed with previous data where the inhibition of PKA decreased NMDA-evoked currents through GluN1/2B NMDA receptors but had no effect on GluN1/2A currents (Skeberdis et al., 2006). To reveal the role of PKA in the age-dependent effects of NaHS the hippocampal slices were incubated with the inhibitor of adenylate cyclase MDL-12330A for 20 min. In these conditions in the slices of newborn animals NaHS induced the decrease of the total charge transfer of NMDA evoked currents from 0.7 ± 0.1 μC to 0.4 ± 0.09 μC (*n* = 9, *N* = 6; *p* = 0.01; Figures 1E,F). At the same time in juvenile animals the increase of NMDA evoked currents was observed by NaHS application from 2.8 ± 0.4 μC to 4.2 ± 0.8 (*n* = 5, *N* = 4; *p* = 0.03; Figures 1E,F). Thus, the inhibition of adenylate cyclase did not prevent the age-dependent effects of NaHS on NMDA evoked currents in pyramidal neurons of hippocampus of newborn and juvenile animals.

### NaHS Induced Opposite Effects on GluN1/2A and GluN1/2B NMDA Evoked Currents Expressed in HEK293T Cells

**Figure 2:**
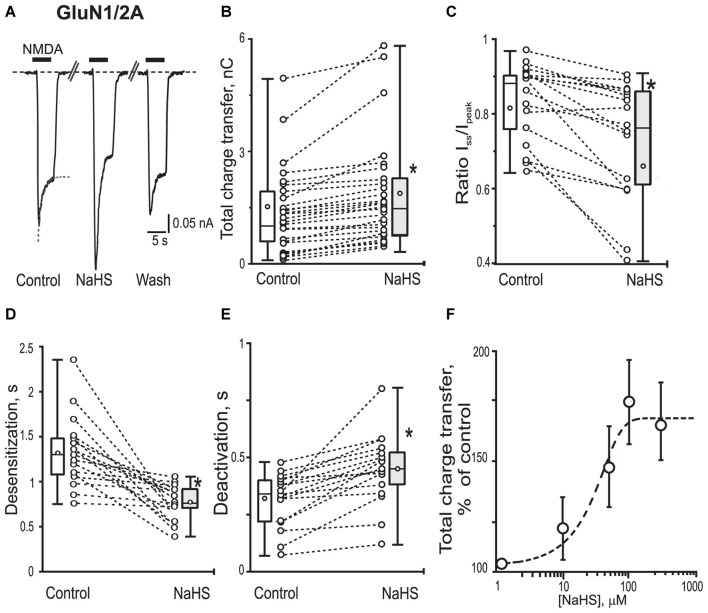
Effects of NaHS on recombinant GluN1/2A containing NMDA receptors expressed in HEK293T cells. Representative current traces recorded in HEK293T cells expressing GluN1/2A activated by application of 100 μM NMDA + 30 μM glycine (5 s, black bar) in control, in the presence of 100 μM NaHS and after washout **(A)**. Dotted lines in control trace represent a fit of a single exponential function to current decay. Statistical plots of the total charge transfer (**B**; *n* = 28), the I_ss_/I_pk_ ratio (**C**; *n* = 17), desensitization time (**D**; *n* = 17) and deactivation time (**E**; *n* = 17) of NMDA evoked currents through GluN1/2A receptors in control and in the presence of NaHS. Each pair of connected circles corresponds to an individual HEK293T cell expressing GluN1/2A. Boxes indicate 25–75 percentiles in control (white) and in NaHS (gray), black line—median, the circle inside—mean value, whiskers—minimal and maximal values. **(F)** Dose dependent effects of NaHS on the total charge transfer in recombinant GluN1/2A receptors. Each point represents the mean value and SEM. Data points were fitted using the dose-response equation (stippled line) with EC_50_ = 49 ± 18 μM (*n* = 6). *p < 0.05, paired *W*-test.

To reveal the role of subunit composition of NMDA receptors in the effect of NaHS recombinant GluN1/2A or GluN1/2B receptors were expressed in HEK293T cells. In HEK cells expressing GluN1/2A receptors application of 100 μM NMDA + 30 μM Gly at a holding potential of −60 mV evoked inward currents with an average peak amplitude of 0.40 ± 0.06 nA (*n* = 28) and a total charge transfer of 1.56 ± 0.2 nC (*n* = 28; Figures 2A,B; Table 1). Preliminary application of 100 μM NaHS for 3 min resulted in potentiation of the amplitude and the area under NMDA induced currents and simultaneously decreased the desensitization time. The peak amplitude of NMDA evoked currents significantly increased up to 140 ± 8%; compared to control (*n* = 28; *p* = 0.0005) and also the charge transfer increased up to 147 ± 8% (*n* = 28; *p* = 0.0002; Figures 2A,B; Table 1). GluN1/2A currents desensitized with an exponential time constant of 1.3 ± 0.1 s (*n* = 20) in control and 0.8 ± 0.07 s in the presence of NaHS (*n* = 17; *p* = 0.004; Figure 2D). The I_ss_/I_pk_ ratio of NMDA mediated currents were reduced from 0.81 ± 0.02 to 0.66 ± 0.04 (*n* = 17; *p* = 0.03; Figure 2C) which indicates acceleration of NMDA receptor desensitization. The deactivation time of NMDA currents in the presence of 100 μM NaHS increased from 0.32 ± 0.05 s to 0.45 ± 0.05 s (*n* = 17; *p* = 0.0003; Figure 2E). Analysis of dose-dependency of NaHS effects revealed an EC_50_ = 49 ± 18 μM (*n* = 6; Figure 2F).

**Table 1:** Sodium hydrosulfide (NaHS) effects on N-methyl-D-aspartate (NMDA) evoked currents in GluN1/2A and GluN1/2B receptors expressed in HEK293T cells.

	Amplitude, nA	Total charge transfer, nC	I_ss_/I_pk_ ratio	Desensitization time, s	Deactivation time, s
**GluN1/2A NMDA receptors**					
Control	0.40 ± 0.06	1.56 ± 0.2	0.81 ± 0.02	1.3 ± 0.1	0.32 ± 0.05
	(*n* = 28)	(*n* = 28)	(*n* = 17)	(*n* = 17)	(*n* = 17)
NaHS	0.52 ± 0.09*	1.83 ± 0.2*	0.66 ± 0.04*	0.8 ± 0.07*	0.45 ± 0.05*
Washout	0.36 ± 0.07	1.44 ± 0.2	0.86 ± 0.08	1.2 ± 0.1	0.39 ± 0.11
**GluN1/2B NMDA receptors**					
Control	0.08 ± 0.009	0.38 ± 0.05	0.91 ± 0.01	1.8 ± 0.2	0.43 ± 0.05
	(*n* = 48)	(*n* = 48)	(*n* = 48)	(*n* = 18)	(*n* = 18)
NaHS	0.06 ± 0.005*	0.26 ± 0.04*	0.89 ± 0.01	1.6 ± 0.2	0.54 ± 0.04*
Washout	0.07 ± 0.01	0.32 ± 0.05	0.92 ± 0.3	1.7 ± 0.4	0.46 ± 0.06

*p < 0.05 compared to control (paired *W*-test).

**Figure 3:**
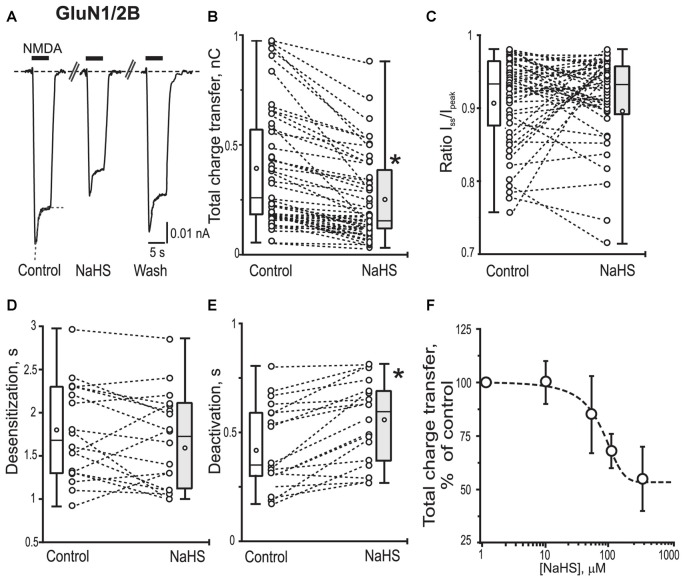
Effect of NaHS on recombinant GluN1/2B containing NMDA receptors expressed in HEK293T cells. Representative current traces recorded in cells expressing GluN1/2B activated by 100 μM NMDA + 30 μM glycine (5 s, black bar) in control, in the presence of NaHS (100 μM) and after washout **(A)**. Dotted lines in control trace represent a fit of a single exponential function to current decay. Statistical plots of the total charge transfer (**B**; *n* = 48), the I_ss_/I_pk_ ratio (**C**; *n* = 48), desensitization time (**D**; *n* = 18) and deactivation time (**E**; *n* = 18) of NMDA evoked currents through GluN1/2B receptors in control and in the presence of NaHS. Each pair of connected circles corresponds to an individual HEK293T cell expressing GluN1/2B. Boxes indicate 25–75 percentiles in control (white) and in NaHS (gray), black line—median, the circle inside—mean value, whiskers—minimal and maximal values. **(F)** Dose dependent effects of NaHS on the total charge transfer in recombinant GluN1/2B receptors. Data points were fitted using the dose-response equation (stippled line) with EC_50_ = 66 ± 15 μM, *n* = 6. *p < 0.05, paired *W*-test.

In recombinant GluN1/2B NMDA receptors application of 100 μM NMDA + 30 μM Gly evoked inward currents with an average peak amplitude of 0.08 ± 0.009 nA and a total charge transfer of 0.38 ± 0.05 nC (*n* = 48; Figures 3A,B; Table 1). Preliminary application of NaHS (100 μM) for 3 min resulted in a decrease of the peak amplitude to 66 ± 5% of control (*n* = 48; *p* = 0.001) and a total charge transfer to 68 ± 8% of control (*n* = 48; *p* = 0.0008; Figures 3A,B; Table 1). The NMDA receptor desensitization rate did not show significant change as indicated by measurements of the decay time and I_ss_/I_pk_ ratio (*n* = 48; *p* = 0.5; Figure 3C; Table 1). The macroscopic desensitization of GluN1/2B currents followed a single exponential time course during NaHS application which was similar to the value measured in control conditions (*n* = 18; *p* = 0.2; Figure 3D; Table 1). The deactivation time of NMDA currents in the presence of NaHS increased from 0.43 ± 0.05 to 0.54 ± 0.04 s (*n* = 18; *p* = 0.001; Figure 3E). Analysis of the dose-dependency of NaHS effects on total energy transfer in GluN1/2B NMDA receptors revealed EC_50_ = 66 ± 15 μM (*n* = 6; Figure 3F).

### The Role of Reduction of Disulfide Bonds in the Effects of NaHS on GluN1/2A and GluN1/2B NMDA Receptors

**Figure 4:**
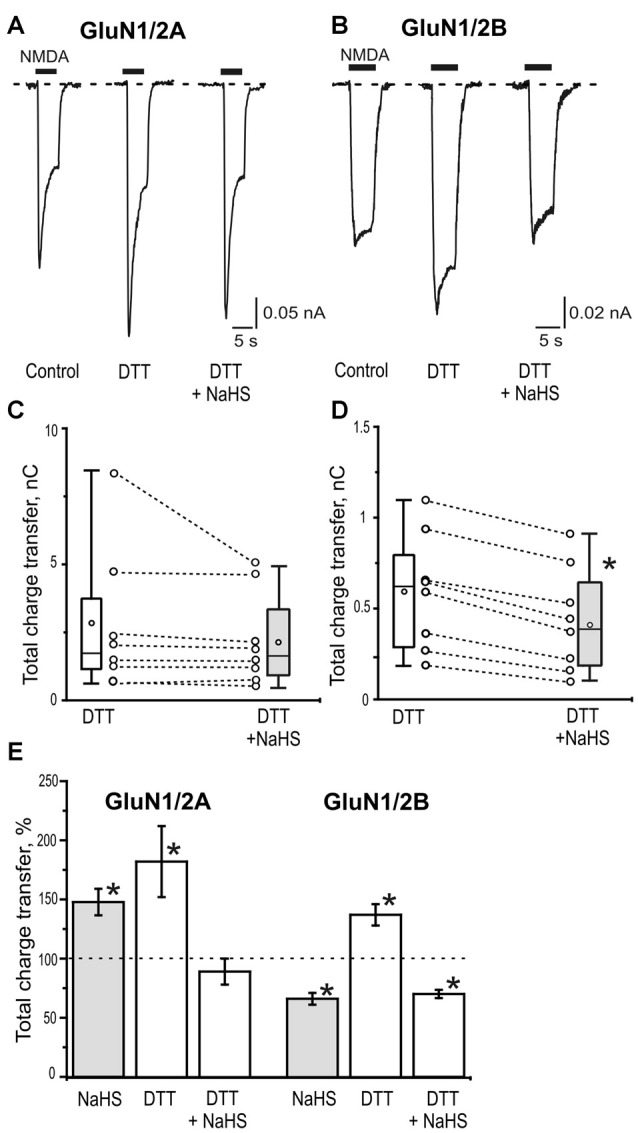
The role of disulfide bonds reduction in the effects of NaHS on GluN1/2A and GluN1/2B receptors. Representative current traces activated by 100 μM NMDA + 30 μM glycine (5 s, black bar) in control, after application of 2 mM dithiothreitol (DTT) and 2 mM DTT+100 μM NaHS in HEK293T cells expressing GluN1/2A **(A)** or GluN1/2B **(B)** receptors. Statistical plot of NMDA induced total charge transfer in the presence of 2 mM DTT and DTT+NaHS. Each pair of connected circles corresponds to an individual HEK293T cell expressing GluN1/2A (**C**; *n* = 8) and GluN1/2B (**D**; *n* = 8). Boxes indicate 25–75 percentiles in control (white) and in NaHS (gray), black line—median, the circle inside—mean value, whiskers—minimal and maximal values, *p < 0.05, paired *W*-test. **(E)** Total charge transfer of NMDA evoked currents in HEK293T cells expressing GluN1/2A (*n* = 8) or GluN1/2B (*n* = 8) receptors after application of NaHS (100 μM) and DTT (2 mM) each normalized to its own control (dotted line) and DTT+ NaHS each normalized to total charge transfer after DTT (dotted line). *p < 0.05 compared to control, paired *W*-test.

H_2_S is known for its reducing action and some of its signaling mechanisms are mediated by the reduction of disulfide bond of channel proteins (Sitdikova et al., 2010; Greiner et al., 2013; Kimura, 2016). The redox modulation of NMDA receptors is one of the known regulatory mechanisms of H_2_S functions (Tang and Aizenman, 1993; Köhr et al., 1994; Lipton and Stamler, 1994). In this mater the reducing agent DTT was used. Bath application of DTT (2 mM) significantly potentiated NMDA induced currents in HEK293T cells expressing GluN2A as well as GluN2B subunits of NMDA receptors (Figures 4A–D; Table 2). The average peak amplitude of NMDA induced currents in DTT-treated cells after 3 min significantly increased from up to 149 ± 7% of control (*n* = 8; *p* = 0.01) in GluN1/2A and up to 154 ± 12% of control (*n* = 8; *p* = 0.01) in GluN1/2B receptors (Table 2). The charge transfer was increased up to 182 ± 30% of control (*n* = 8; *p* = 0.01; Figures 4A,C) for GluN1/N2A and up to 137 ± 9% of control (*n* = 8; *p* = 0.01) for GluN1/N2B receptors (Figures 4B,D; Table 2). At the same time DTT induced a reduction of the desensitization time for GluN1/2A NMDA receptors (I_ss_/I_peak_ 0.78 ± 0.02 in control vs. 0.65 ± 0.07 in DTT, *n* = 8; *p* = 0.02) but had no effect on GluN1/2B NMDA receptor desensitization (*n* = 10; *p* = 0.06; Table 2). The macroscopic desensitization of GluN1/2A current in DTT-treated cells was reduced from 1.2 ± 0.05 s to 0.6 ± 0.1 s (*n* = 8; *p* = 0.04) and did not change in GluN1/2B receptors (*n* = 10; *p* = 0.21; Table 2). The time of receptor deactivation after DTT incubation for GluN1/2A increased from 0.32 ± 0.05 to 0.55 ± 0.08 s (*n* = 8; *p* = 0.01) and for GluN1/2B-from 0.43 ± 0.05 to 0.78 ± 0.08 s (*n* = 8; *p* = 0.007; Table 2).

**Table 2:** The role of reduction of disulfide bonds in the effects of NaHS on GluN1/2A and GluN1/2B NMDA receptors.

	Amplitude, nA	Total charge transfer, nC	I_ss_/I_pk_ ratio	Desensitization time, s	Deactivation time, s
**GluN1/2A NMDA receptors**					
Control	0.29 ± 0.09	1.1 ± 0.5	0.78 ± 0.02	1.2 ± 0.1	0.32 ± 0.05
	(*n* = 8)	(*n* = 8)	(*n* = 8)	(*n* = 8)	(*n* = 8)
DTT	0.42 ± 0.13*	2.6 ± 1.6*	0.65 ± 0.07*	0.6 ± 0.1*	0.55 ± 0.08*
DTT+NaHS	0.36 ± 0.12	2.0 ± 0.6	0.66 ± 0.09	1.1 ± 0.1	0.61 ± 0.11
**GluN1/2B NMDA receptors**					
Control	0.11 ± 0.02	0.45 ± 0.1	0.91 ± 0.08	1.7 ± 0.3	0.43 ± 0.05
	(*n* = 8)	(*n* = 8)	(*n* = 8)	(*n* = 10)	(*n* = 8)
DTT	0.15 ± 0.01*	0.58 ± 0.11*	0.93 ± 0.01	1.6 ± 0.2	0.78 ± 0.08*
DTT+NaHS	0.11 ± 0.02^#^	0.44 ± 0.1^#^	0.92 ± 0.03	1.5 ± 0.2	0.82 ± 0.1

*p < 0.05 compared to control; ^#^p < 0.05 compared to dithiothreitol (DTT; paired W-test).

In DTT-treated cells subsequent application of NaHS did not induce an increase of NMDA evoked current amplitudes (87 ± 12% of DTT response; *n* = 8; *p* = 0.09) and charge transfer of GluN1/2A receptors (89 ± 7% of DTT response; *n* = 8; *p* = 0.25; Figures 4A,C,E; Table 2). The time course of desensitization and rate of receptors deactivation did not change significantly (Table 2).

In contrast to GluN1/2A in GluN1/N2B receptors application of NaHS still decreased the average peak amplitude and total charge transfer to 73 ± 5% of DTT responses; *n* = 8; *p* = 0.008) and 70 ± 4% of DTT responses, *n* = 8; *p* = 0.003) respectively (Figures 4B,D,E; Table 2). The time course of desensitization and the rate of receptor deactivation did not significantly change (*n* = 10; *p* = 0.78 and 0.6, correspondingly; Table 2).

## Discussion

The main finding of our study is that effects of H_2_S on NMDA currents are age-dependent in rat hippocampus. We found that, in neonatal rats, H_2_S decreased whereas in juvenile rats potentiated NMDA evoked currents in hippocampal pyramidal neurons and these effects were not prevented by the inhibition of adenylate cyclase. We also showed that NaHS decreased currents via NMDA receptors composed by recombinant GluN1/2B subunits but increased currents through GluN1/2A NMDA receptors providing a mechanistical explanation for the age-dependent action of H_2_S in brain slices. The reducing agent DTT selectively prevented effects of NaHS on GluN1/2A receptors not affecting the inhibitory action of NaHS on GluN1/2B receptors.

### Age-Dependent Effects of H_2_S on NMDA Receptor Mediated Currents in Rat Hippocampal Neurons

First evidence of the neuromodulatory role of H_2_S in rat hippocampus was obtained by Abe and Kimura (Abe and Kimura, 1996). They found that the donor of H_2_S NaHS suppressed the excitatory postsynaptic potentials in the CA1 region of hippocampus. H_2_S-producing enzymes CBS, 3-MST/CAT and DAO are highly expressed in the hippocampus (Abe and Kimura, 1996; Enokido et al., 2005; Kimura, 2010; Renga, 2011; Bruintjes et al., 2014). The activity of these enzymes is sensitive to different modulators such as *S*-adenosyl methionine (SAM), nitric oxide, carbon monoxide and Ca^2+^ (Kimura, 2010). In our previous study we showed that H_2_S induced the biphasic effects on spontaneous neuronal activity in the neonatal rat hippocampus with an initial increase followed by inhibition of network-driven GDPs and multiple unit activity (Yakovlev et al., 2017). In the present study, we further investigated the role of NMDA receptors in the action of H_2_S. Notably, we found that NMDA receptor mediated currents were modulated by H_2_S in the opposite direction, dependent of the animal age. Specifically, we demonstrated that in neonatal slices NaHS decreased NMDA responses, whereas in older animals (2–3 weeks old) we found a significant increase of NMDA evoked currents. These findings are consistent with NaHS-induced increase of NMDA currents in the hippocampus in 2–3 week-old rats (Abe and Kimura, 1996).

A developmental turnover of GluN2B to GluN2A containing NMDA receptors is recognized as common feature for many areas in the brain (Liu et al., 2004; van Zundert et al., 2004; Zhou and Baudry, 2006). In both the cortex and the hippocampus, GluN2B subunit dominates in early development and slowly declines as neurons mature. In contrast, the GluN2A subunit is increasingly expressed during development and becomes dominant in adult neurons (Li et al., 1998; Chang et al., 2009). This developmental shift could explain the age-dependent effects of H_2_S on NMDA evoked currents. Similar results were observed for SH-group containing amino acid homocysteine which reduced peak amplitudes of NMDA currents in premature neurons and in HEK293T GluN2B-expressed cells (Bolton et al., 2013). Conversely homocysteine increased NMDA currents in GluN2A expressed HEK293T cells (Bolton et al., 2013; Sibarov et al., 2016).

It is known that PKA modulates NMDA receptor function and NMDA receptor-dependent synaptic plasticity (Skeberdis et al., 2006; Lau et al., 2009). Moreover the inhibition of PKA differentially regulates NMDA evoked currents through NMDA receptors of different subunit composition. Inhibition of PKA decreased NMDA-evoked currents through GluN1/2B NMDA receptors but had no effect on GluN1/2A currents (Skeberdis et al., 2006).

In early studies H_2_S has been demonstrated that it is able to increase cAMP production in primary neurons from cerebral cortex and cerebellum (Kimura, 2000), which may mediate positive effects of NaHS effects on NMDA receptors. From the other hand forskolin, an activator of adenylyl cyclase did not significantly increase NMDA currents in hippocampal neurons (Skeberdis et al., 2006), suggesting that under control conditions, the concentration of cAMP is not rate-limiting for PKA-mediated phosphorylation. In our experiments incubation of brain slices in the inhibitor of adenylate cyclase did not prevent the increase of NMDA-evoked currents in juvenile animals, which is in agreement with the absence of the effect of PKA inhibition on NMDA-evoked currents through GluN1/2A receptors (Skeberdis et al., 2006). In case of newborn animals we still observed the decrease of NMDA-evoked currents after application of NaHS, which indicates on PKA-independent mode of H_2_S action on GluN1/2B NMDA receptors. Moreover the effects of H_2_S on cAMP production are varied among different cell types or tissues and may be secondary to other signaling pathways (e.g., COX-2 or phosphodiesterase; Njie-Mbye et al., 2012; Perniss et al., 2017). Whereas the study of Kimura (2000) provides evidence for an activation of the cAMP-pathway by H_2_S, inhibition of cAMP-concentration has also been reported (Yong et al., 2008; Nagpure and Bian, 2014; Yang et al., 2014). Therefore, the effect of H_2_S on cAMP production may be influenced by many factors, which may include different cell types, cellular targets and their activity status, adenylate cyclase and phosphodiesterase isoforms, treatment period with H_2_S and activation of other signaling pathways in different pathological situations.

### The Role of Subunit Specificity and the Reducing Action of H_2_S

Subunit specificity of H_2_S action was established in our experiments by using recombinant GluN1/2A and GluN1/2B subunit containing NMDA receptors expressed in HEK293T cells. NaHS induced potentiation of GluN1/2A receptor mediated currents but inhibition of GluN1/2B receptor mediated currents. H_2_S also accelerated desensitization of GluN1/2A NMDA receptors and reduced deactivation rate of both GluN1/2A and GluN1/2B receptors. The activating effect of H_2_S was previously shown on GluN1/2A NMDA receptors expressed in *Xenopus* oocytes, where NaHS decreased the onset time of NMDA responses (Kimura, 2010).

As NMDA receptors are highly redox sensitive we also analyzed the role of disulfide bonds reduction in the effects of H_2_S. The reducing agent DTT potentiated NMDA activated currents, decreased deactivation rate in both GluN1/2A and GluN1/2B receptors, and accelerated desensitization of GluN1/2A receptors consistent with previously published data (Aizenman et al., 1989; Köhr et al., 1994). Interestingly, DTT was able to prevent selectively the action of NaHS on GluN1/2A receptors. However, the inhibitory action of H_2_S on GluN1/2B receptors in the presence of DTT remained the same. These differences can be explained by the presence of distinct redox sensitive sites in GluN1/2A and GluN1/2B receptors (Aizenman et al., 1989; Köhr et al., 1994; Sullivan et al., 1994). In GluN1/2A receptors the DTT-sensitive sites are located at extracellular terminal and in the ligand binding domain of the GluN1 subunit (Talukder et al., 2011). In NMDA receptor containing GluN2B subunit the redox sites are located in the extracellular loop between the transmembrane domains III and IV of GluN1 subunit (Sullivan et al., 1994) but also in GluN2B subunit (Brimecombe et al., 1999). Interestingly, similar to NaHS, mercaptoethylamine and the membrane-impermeable endogenous reducing agent glutathione potentiated only GluN1/2A receptors (Köhr et al., 1994). The conformation of NMDA receptors could also play a role in the access of reducing agents to disulfide bonds (Brimecombe et al., 1997). These peculiarities of redox modulation of NMDA receptors of different subunit composition may explain the opposite effect of H_2_S on GluN1/2A and GluN1/2B NMDA receptors.

### Physiological and Pathophysiological Significance

Subunit-specific action of H_2_S on NMDA receptor mediated currents appears to be important in early development and in aging. Notably, the functional role of NMDA receptors strongly depends on their localization. Thus, the GluN1/2A containing NMDA receptors are mainly localized at synaptic sites to be activated by glutamate released from the presynaptic terminals. In contrast, GluN2B subunit containing NMDA receptors are preferentially localized at extrasynaptic somatic and dendritic sites, being activated by glutamate spillover, especially pronounced in stroke and brain trauma (Rossi et al., 2007; Harris and Pettit, 2008). Importantly, GluN2B subunits are involved in NMDA induced excitotoxicity and apoptosis (Hardingham and Bading, 2010; Zhou et al., 2015). Therefore, the inhibition of extrasynaptic GluN1/N2B receptors by H_2_S could be involved in its neuroprotective action against glutamate excitotoxicity (Eghbal et al., 2004; Kimura and Kimura, 2004).

Activation of synaptic GluN2A subunit containing NMDA receptor could underlie the supportive effects of H_2_S on synaptic plasticity and induction of LTP (Abe and Kimura, 1996) and appears to have protective role in cognitive decline during aging and neurodegenerative disorders. Indeed, redox changes and decrease in NMDAR function contribute to senescent synaptic function in vulnerable brain regions involved in age-related cognitive decline (Guidi et al., 2015). Moreover the impaired LTP in hippocampal slices induced by aging could be reversed by acute administration of reductants, such as DTT or β-mercaptoethanol and was mimicked by glutathione (Bodhinathan et al., 2010; Yang et al., 2010; Kumar and Foster, 2013). Notably, the level of endogenous H_2_S is severely decreased in brains of Alzheimer’s disease (AD) patients (Eto et al., 2002; Liu and Bian, 2010) and in a rat model of Parkinson’s disease (PD; Hu et al., 2010). In experimental models of AD and PD H_2_S have been shown to attenuate the decline of learning and memory, oxidative stress and neuroinflammation (Liu and Bian, 2010; Kida et al., 2011; Giuliani et al., 2013). These data propose that H_2_S is effective against neurodegeneration and neurovascular dysfunctions (Kamat et al., 2013). Indeed, the restoring of the thiol redox status may be an effective strategy in the treatment of oxidative injury. Recently published data emphasizes the protective effects of H_2_S against spatial memory retrieval impairment caused by acute stress (He et al., 2017). Moreover, the therapeutic potential of H_2_S was suggested in the treatment of brain ischemia, where H_2_S significantly improved spatial learning, memory deficits and enhanced synaptic plasticity in the hippocampus of brain-ischemic rats (Li et al., 2011).

In the developing brain the inhibitory action of H_2_S on GluN1/2B NMDA receptors could limit the excessive neuronal excitability typical to early hippocampal networks (Ben Ari et al., 2012; Marutani et al., 2012). Indeed, H_2_S has been found to eliminate the interictal-like events induced by bicuculline in neonatal hippocampus (Yakovlev et al., 2017). The expression of CBS in the hippocampus and the plasma level of H_2_S were dramatically increased in rat model of recurrent febrile seizure which may indicate a compensatory response to neuronal hyper-excitability (Han et al., 2005). In this regard, H_2_S could contribute to control of the excitatory/inhibitory balance in the developing brain. In fact, the expression of the H_2_S-producing enzymes CBS and 3-MST increases from the late embryonic to the early postnatal period (Enokido et al., 2005) highlighting the importance of this signaling pathway. Apart from the production of H_2_S, another important role of CBS may comprise a decrease of the neurotoxic aminoacid homocysteine level in early development (Rosenquist and Finnell, 2001; Gerasimova et al., 2017).

In conclusion, the functionally opposite age-dependent effects of H_2_S on NMDA mediated currents are determined by glutamate NMDA receptor subunit composition. The action of H_2_S could provide the neuroprotection against hyperexcitability in the immature brain and participate in prevention of the cognitive decline during aging.

## Author Contributions

AVY, RG and GFS designed experiments; wrote the article. AVY, EDK and YI performed the experiments. AVY and EDK analyzed the data.

## Conflict of Interest Statement

The authors declare that the research was conducted in the absence of any commercial or financial relationships that could be construed as a potential conflict of interest.

## Abbreviations

AMPA: α-amino-3-hydroxy-5-methyl-4-isoxazolepropionic acid
CAT: cysteine aminotransferase
CBS: cystathionine β-synthase
CNQX: 6-cyano-7-nitroquinoxaline-2,3dione
CSE: cystathionine γ-lyase (CSE)
DAO: D-amino acid oxidase
d-APV: d-2-Amino-5-phosphopentanoate
DTT: dithiothreitol
H_2_S: hydrogen sulfide
3-MST: 3-mercaptopyruvate sulfurtransferase
NaHS: sodium hydrosulfide
NMDA: N-methyl-D-aspartate.
